# Long-term outcomes of platinum-based chemotherapy for T4 stage sinonasal adenoid cystic carcinoma

**DOI:** 10.3389/fphar.2025.1623242

**Published:** 2025-09-29

**Authors:** Xinmao Song, Ji Sun, Gang Yang, Xiaoshen Wang, Li Wang

**Affiliations:** ^1^ Department of Radiotherapy, Eye & ENT Hospital, Fudan University, Shanghai, China; ^2^ Department of Pathology, Eye & ENT Hospital, Fudan University, Shanghai, China

**Keywords:** sinonasal adenoid cystic carcinoma, T4 stage, platinum-based, chemotherapy, long-term survival

## Abstract

**Purpose:**

To investigate the impact of platinum-based chemotherapy on long-term outcomes of T4 sinonasal adenoid cystic carcinoma (SNACC).

**Methods:**

We retrospectively analyzed clinical data from 87 consecutive patients with T4 SNACC who underwent surgery plus radiotherapy with or without platinum-based chemotherapy in our institution between 1993 and 2019.

**Results:**

Of the 87 cases of T4 SNACC, 57.5% (50/87) of patients underwent platinum-based chemotherapy. Overall, chemotherapy was not associated with overall survival (OS) (P = 0.814), PFS (progression-free survival) (P = 0.508), local recurrence-free survival (LRFS) (P = 0.500) or distant metastasis-free survival (DMFS) (P = 0.202) improvement. Besides, chemotherapy significantly decreased 10-year OS (27.9% vs. 58.8%, P = 0.034) compared with the no-chemotherapy group. Univariate (P = 0.383) and multivariate logistic analyses (P = 0.213) suggested that chemotherapy did not reduce the risk of distant metastasis. Furthermore, multivariate Cox regression analysis indicated that chemotherapy did not improve OS (P = 0.789) or PFS (P = 0.556).

**Conclusion:**

Platinum-based chemotherapy did not seem to provide a long-term survival advantage for patients with T4 SNACC and might be associated with worse outcomes after 5 years, although these findings need confirmation through prospective studies.

## Introduction

Adenoid cystic carcinoma (ACC) is the second most common histologic subtype of sinonasal malignancies after squamous cell carcinoma (SCC), accounting for 5%–15% of malignant paranasal sinus tumors (Husain et al., 2013; Dulguerov et al., 2001). It is characterized by slow growth, a tendency for perineural invasion, late recurrence, and distant metastasis (Bradley, 2004; Chummun et al., 2001). More than 50% of cases show notable invasiveness with a propensity for distant metastasis, commonly to the lungs, bones, and livers, ultimately resulting in low survival rates (Seok et al., 2019; Cavalieri et al., 2020). Given its slow-growing biological nature and non-specific symptoms in the early stage, the diagnosis of sinonasal adenoid cystic carcinoma (SNACC) is often delayed and usually found in advanced stages (65%–76% T3-T4 tumors). A meta-analysis involving 17 studies with 2,300 patients showed that high rates of locally advanced tumor stages at diagnosis: 23% cT3, 53% cT4 (Mauthe et al., 2023). This leads to a poorer prognosis compared with other head and neck ACCs, such as salivary gland counterparts (Michel et al., 2013).

The optimal treatment consists of surgical resection and postoperative adjuvant radiotherapy for SNACC patients (Husain et al., 2013; Rhee et al., 2006; Wiseman et al., 2002). Platinum-based single-agents and combination regimens are usually administered to advanced diseases, including unresectable, recurrent or metastatic tumors, while limited data on the effectiveness of these treatments are available, mainly due to the indolent course of the disease and the lack of large-sized studies or prospective trials to evaluate the effect of chemotherapy (Papaspyrou et al., 2011; Laurie et al., 2011) (Rentschler et al., 1977; Dodd and Slevin, 2006; Vermorken et al., 1993; Ross et al., 2009). At present, cisplatin, doxorubicin, cyclophosphamide (CAP) was the most studied regimen in ACC, achieving an objective response rate of 25% (Licitra et al., 1996). Therefore, the value of chemotherapy in reducing distant metastasis and improving long-term survival remain controversial for ACC patients (Coca-Pelaz et al., 2015).

Due to the rarity of the disease, very few clinical reports have investigated the effect of chemotherapy in managing SNACC, and its impact on distant metastasis and long-term survival remains unclear. Moreover, the clinical usefulness of chemotherapy in patients with SNACC has been a topic of debate because of the absence of high-level evidence. Additionally, patients with stage T4 lesions exhibit significantly worse overall survival (OS) compared with those with T1-T3 disease in SNACC (Lupinetti et al., 2007). Given the urgent need to improve the OS for stage T4 SNACC, it is essential to explore treatment strategies that include chemotherapy within a multidisciplinary framework. The primary goal of this study was to determine whether chemotherapy could reduce the risk of distant metastasis and improve the long-term survival outcomes for patients with T4-stage SNACC.

## Methods

### Patient selection

A retrospective review was conducted of 87 patients treated at Eye and ENT Hospital, Fudan University, from April 1993 to November 2019. The diagnosis was confirmed by pathological examination. At the time of presentation, all patients in this study had T4 classification disease without distant metastasis. Clinical data regarding patient characteristics, imagings, treatment modalities, treatment outcomes, and follow-up were collected from the medical records. The studies involving human participants were reviewed and approved by the Ethics Committee of Eye and ENT Hospital, Fudan University (approval number 2021033-1). All patients provided informed consent, permitting the use of treatment data for research purposes and statistical analysis. The TNM (tumor, nodes, and metastases) staging method of the AJCC (eighth American Joint Committee on Cancer) was utilized to stage the disease.

### Treatment and analysis

All the patients underwent surgery and radiotherapy. The prescribed radiation dose to the planning target volume (PTV) of the primary site and metastatic lymph nodes was 66–74 Gy delivered in 30–35 fractions, while the dose for the PTV of the high-risk clinical target volume ranged from 58 to 64 Gy. Chemotherapy typically comprised 2 to 5 cycles, predominantly using cisplatin at a dose of 60–75 mg/m^2^ administered every 3 weeks. Whether to administer chemotherapy mainly depends on the patient’s physical condition, age, and the size of the tumor.

### Follow-up

Treatment efficacy was assessed through a complete physical examination and imaging evaluation, including magnetic resonance imaging (MRI) or computed tomography (CT) scan of the head and neck, chest CT scan, and abdominal ultrasound 2–3 months after radiotherapy. The follow-up schedule was performed as follows: every 3–4 months during the first 2 years, every 6 months for the subsequent 3 years, and annually thereafter. Each follow-up visit included a detailed physical examination and imaging studies, which may have included the contrast-enhanced MRI, chest CT scan, abdominal ultrasound, and PET/CT scan if necessary. Follow-up data were collected through routine clinic visits or direct communication with the referring physician or family members. The median follow-up time after treatment was 71.2 months.

### Statistical analysis

Chi-square tests were used to compare demographic features between groups. Multivariate survival analyses were conducted using Cox regression in SPSS statistical software version 26.0^®^ (IBM, New York, United States) to identify factors associated with OS and PFS. Survival probabilities at 5, 10, and 15 years were estimated using Kaplan-Meier curves. Landmark analyses were performed to assess outcomes during the first 5 years and between 5 years and 15 years (Maeng et al., 2014). The differences in survival between groups were analyzed to hazard ratios (HRs) calculate 95% confidence intervals (CIs). Statistical significance was defined as P < 0.05.

## Results

### Patient characteristics

This research included 87 individuals diagnosed with T4 stage SNACC, comprising 49 males and 38 females. The baseline characteristics of these patients were summarized in Table 1. The median age of patients was 55 years, ranging from 14 to 75 years. Notably, the maxillary sinus (65.5%, 57/87) was the most frequently involved site. Only seven patients (8.0%, 7/87) exhibited nodal metastasis, and no distant metastasis was observed at the time of diagnosis. Additionally, fifty patients (57.5%, 50/87) underwent cisplatin-based chemotherapy treatment. There were no significant differences in age, sex, original site or lymph node status between the chemotherapy and the no chemotherapy groups. No statistical differences of patient distribution were also observed in the subgroups with orbital involvement (P = 0.093), nasopharyngeal involvement (P = 0.412), and Cavernous sinus involvement (P = 0.071). While, the trend suggested that the chemotherapy was more often delivered in these subgroups which had a higher burden of high-risk features. Details of the chemotherapy regimens were presented in Supplementary Table S1.

**TABLE 1:** Patients’ clinical characteristics.

Characteristic	Overall	No chemo	Chemo	P -value
	n = 87	n = 37	n = 50	
Age, years				
Median (range), years	55 (14–75)			*p* = 0.762
<60 (%)	62 (71.3)	27 (73.0)	35 (70.0)	
≥60 (%)	25 (28.7)	10 (27.0)	15 (30.0)	
Gender (%)				*p* = 0.421
Male	49 (56.3)	19 (51.4)	30 (60.0)	
Female	38 (43.7)	18 (48.6)	20 (40.0)	
Site (%)				*p* = 0.139
Maxillary sinus	57 (65.5)	21 (56.8)	36 (72.0)	
Nasal cavity/sphenoid/Ethmoid sinus	30 (34.5)	16 (43.2)	14 (28.0)	
T stage (%)				*p* = 0.891
T4a	36 (41.4)	15 (40.5)	21 (42.0)	
T4b	51 (58.6)	22 (59.5)	29 (58.0)	
N status (%)				*p* = 0.985
N-	80 (92.0)	34 (91.9)	46 (92.0)	
N+	7 (8.0)	3 (8.1)	4 (8.0)	
Orbital involvement (%)				*p* = 0.093
No	38 (43.7)	20 (54.1)	18 (36.0)	
Yes	49 (56.3)	17 (45.9)	32 (64.0)	
Nasopharyngeal involvement (%)				*p* = 0.412
No	65 (74.7)	26 (70.3)	39 (78.0)	
Yes	22 (25.3)	11 (29.7)	11 (22.0)	
Cavernous sinus Involvement (%)				*p* = 0.071
No	67 (77.0)	32 (86.5)	35 (70.0)	
Yes	20 (23.0)	5 (13.5)	15 (30.0)	

## Long-term outcomes

Survival data were shown in Supplementary Figure S1. The median follow-up duration was 71.2 months, with a range of 3.7–353.4 months. Among the 87 patients, 45 (51.7%) experienced treatment failures, which included local recurrence in 22 cases (25.3%), distant metastasis in 13 cases (14.9%) or both failures in 10 cases (11.5%). Additionally, 33 (37.9%, 33/87) patients ultimately died of the disease. The OS rates for all the patients’ 5-, 10-, and 15-year were 74.3%, 47.5%, and 33.0%, respectively. Similarly, the PFS rates at the same time points were 60.6%, 31.9%, and 22.8%, respectively.

Multivariate Cox regression analysis revealed that the lymph node status was an independent prognostic factor for worse PFS (HR = 3.844, 95% CI: 1.252–11.801, P = 0.019) in T4 stage SNACC (Table 2). Furthermore, chemotherapy failed to significantly affect either OS (HR = 0.902, 95% CI: 0.425–1.917, P = 0.789) or PFS (HR = 1.212, 95% CI: 0.638–2.302, P = 0.556).

**TABLE 2:** Multivariate analyses of factors in relation to OS and PFS using the Cox proportional hazards model for T4 patients (n = 87).

Variables	OS			PFS		
	HR	95% CI	P	HR	95% CI	P
Age (≥60 vs.<60)	1.549	0.732–3.276	0.253	0.995	0.970–1.020	0.684
Gender (Female vs. Male)	0.995	0.467–2.119	0.990	1.044	0.568–1.919	0.890
Site (Maxillary sinus vs. Nasal/ethmoid sinus	1.157	0.515–2.600	0.724	1.704	0.834–3.481	0.143
Cervical LN (N+ vs. N-)	3.013	0.833–10.896	0.093	3.844	1.252–11.801	**0.019**
Chemo (Yes vs. No)	0.902	0.425–1.917	0.789	1.212	0.638–2.302	0.556
Orbit involvement (Yes vs. No)	1.525	0.642–3.623	0.339	1.420	0.696–2.898	0.335
Nasopharyngeal involvement (Yes vs. No)	1.577	0.722–3.444	0.253	1.944	0.970–3.894	0.061
Cavernous sinus involvement (Yes vs. No	1.248	0.462–3.376	0.662	0.953	0.407–2.230	0.912

OS, overall survival; PFS, Progression-free survival; HR, hazard ratio; LN, lymph node; Chemo, chemotherapy. Values in bold represent statistically significant differences.

**FIGURE 1:**
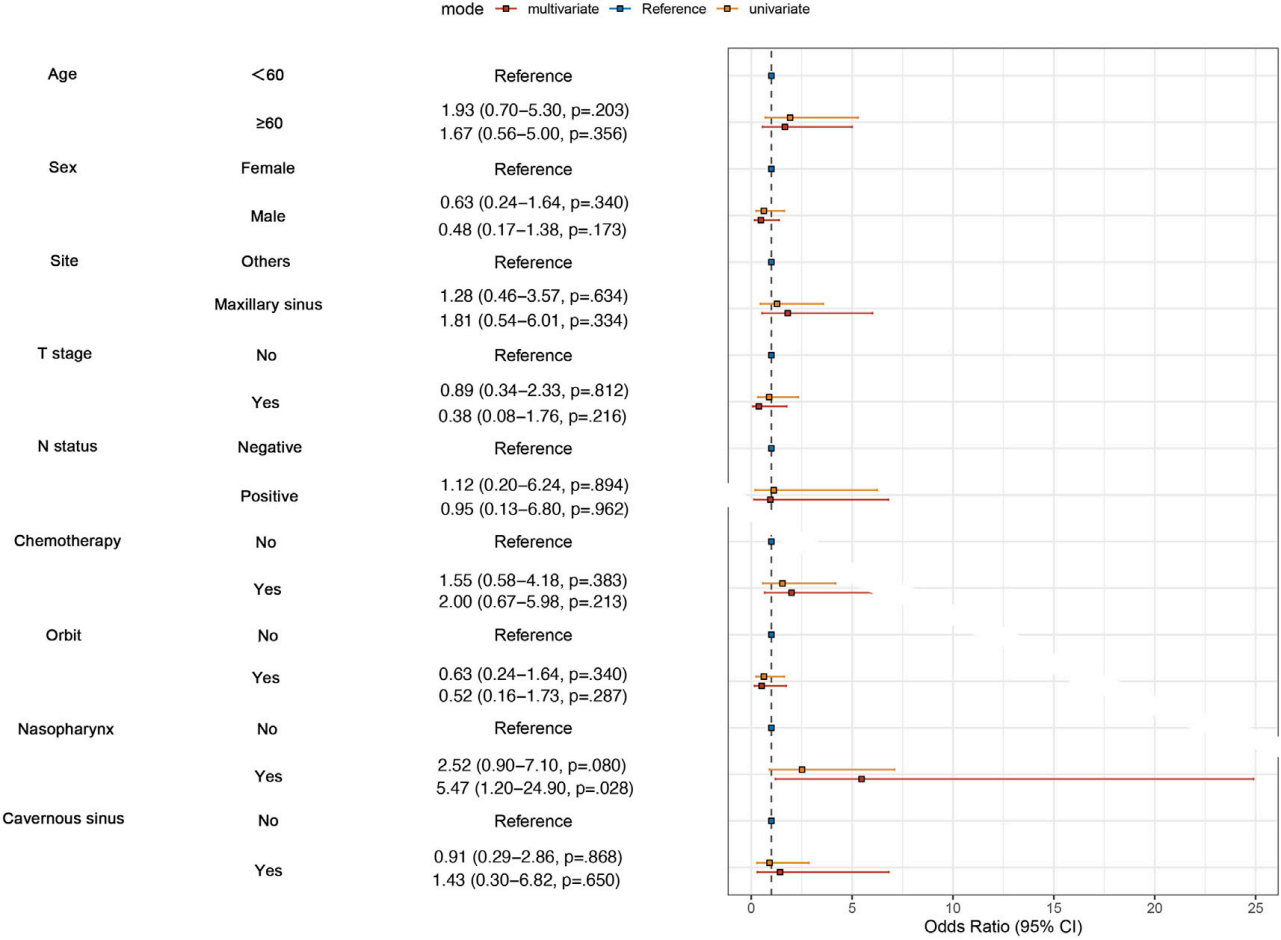
The logistic regression analysis on potential factors of distant metastasis.

To examine whether chemotherapy can reduce the risk of distant metastasis or not in SNACC patients, we performed univariate and multvariate logistic regression analyses. We enrolled clinical factors that were potentially related to metastasis, such as T stage, N stage, and anatomical sites. The results of the multivariate analysis revealed that patients with nasopharyngeal invasion were more likely to develop distant metastasis (P = 0.028), while chemotherapy did not reduce the risk of distant metastasis in both univariate (P = 0.383) and multivariate analyses (P = 0.213) (Figure 1).

### Subgroup analyses

**FIGURE 2:**
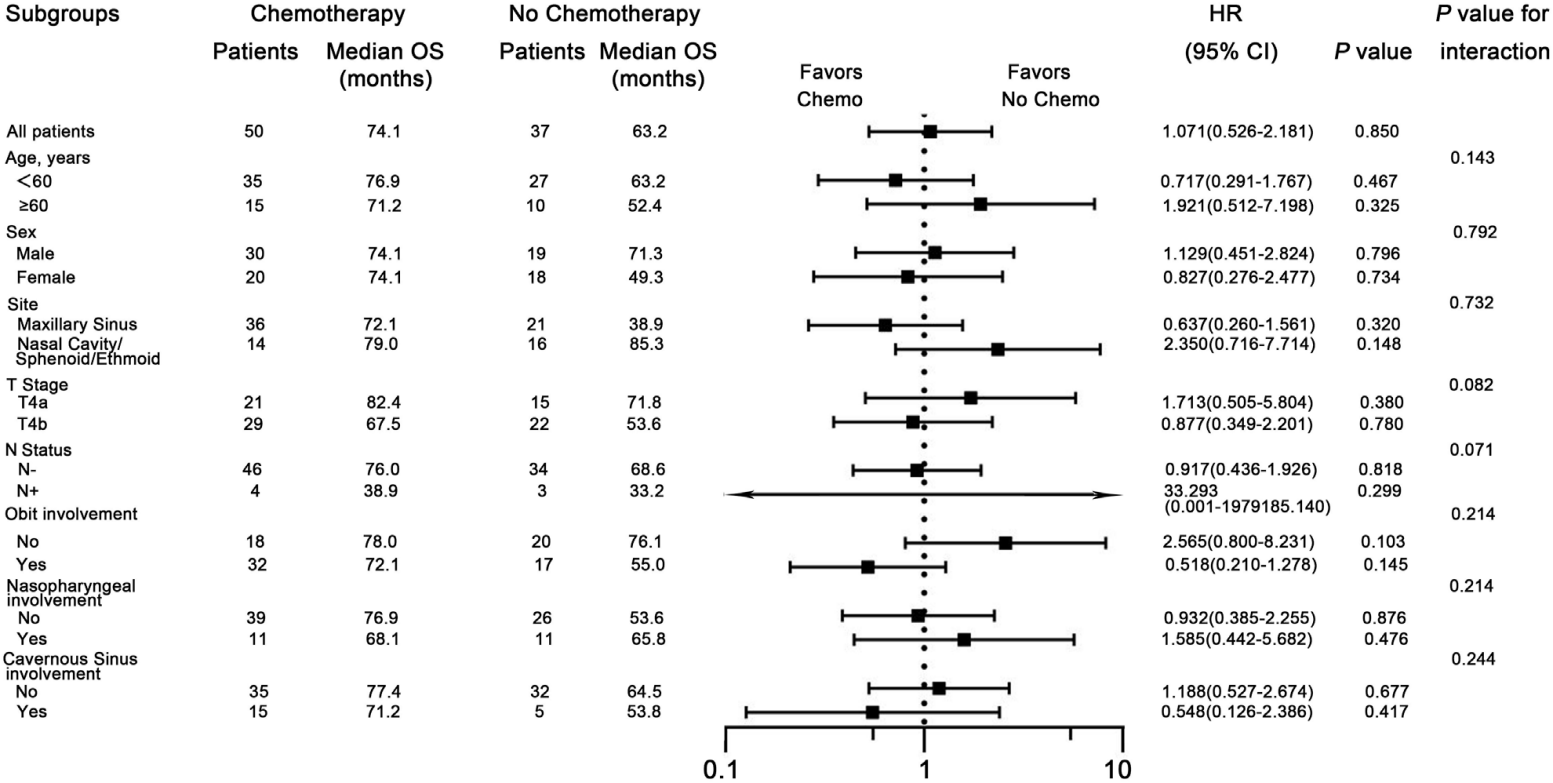
Subgroup analyses for overall survival (OS), comparing patients with and without chemotherapy within subgroups. HR means Hazard Ratio.

Univariate Cox regression analysis was conducted to determined which populations might benefit from chemotherapy. The chemotherapy group demonstrated no OS benefit compared with the no-chemotherapy group (HR = 1.071; P = 0.850). To explore potential interactions between treatment effects and baseline characteristics, subgroup analyses were carried out among patients stratified by sex (male vs. female), age (<60 vs. ≥60 years), tumor site (Maxillary sinus vs. Nasal cavity/sphenoid/ethmoid sinus), T stage (T4a vs. T4b), N status (N- vs. N+), orbital invasion (Yes vs. No), nasopharyngeal invasion (Yes vs. No), and cavernous sinus invasion (Yes vs. No). Forest plots revealed no significant interactions between these covariates and treatment assignment (chemotherapy or not) (Figure 2), indicating that among the common clinical risk factors, no subgroup of common clinical rick factors benefits from chemotherapy.

### The influence of chemotherapy on prognosis

**FIGURE 3:**
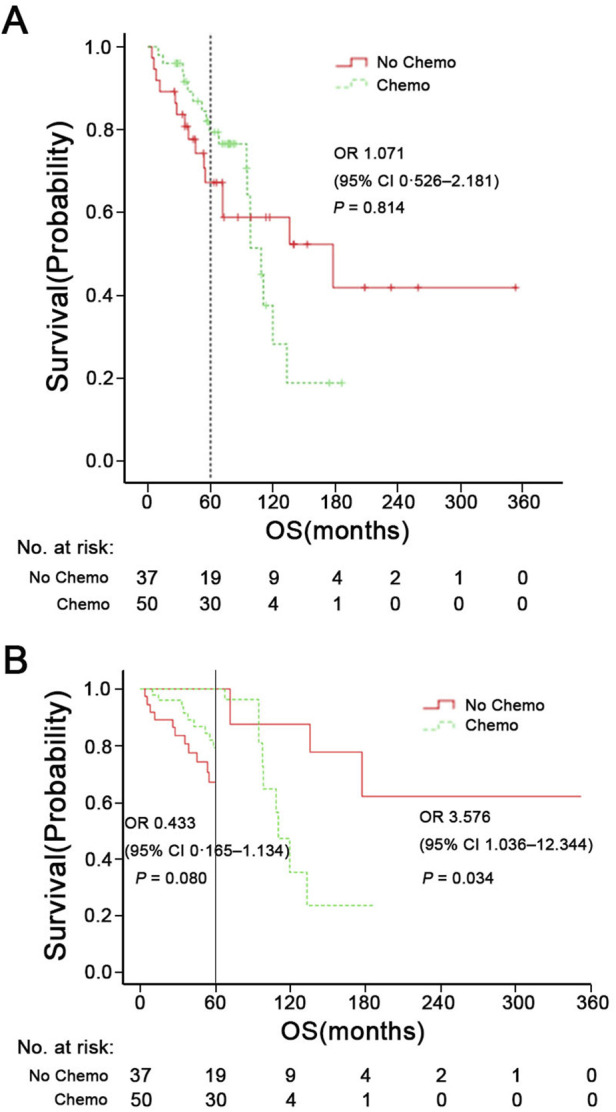
**(A)** Overall survival (OS) of patients with chemotherapy or not. **(B)** Landmark analysis comparing overall survival before and after 5 years of follow-up. HR means Hazard Ratio.

**FIGURE 4:**
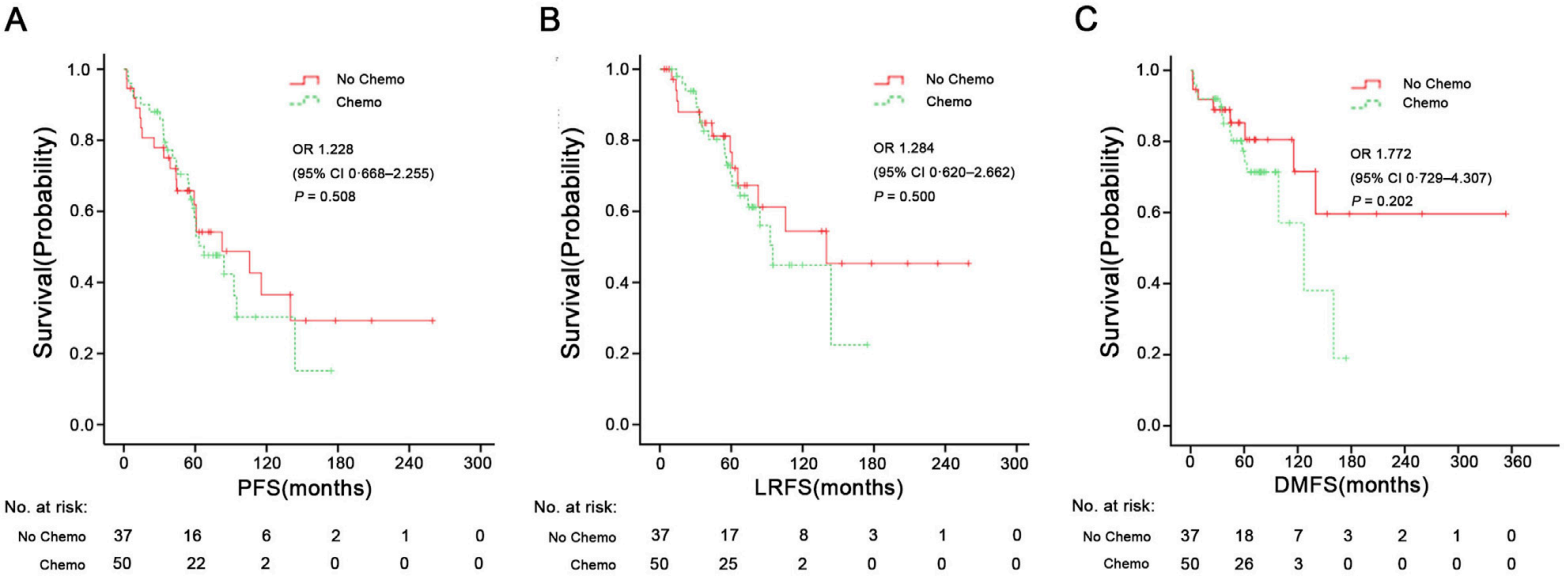
PFS **(A)**, LRFS **(B)**, and DMFS **(C)** in the chemotherapy group and no chemotherapy group. PFS, progression-free survival; LRFS, local recurrence-free survival; DMFS, distant metastasis-free survival. HR, Hazard Ratio.

Figures 3A, 4 shows Kaplan-Meier curves for OS, PFS, LRFS, and DMFS based on whether chemotherapy was administered. Chemotherapy demonstrated no significant impact on long-term prognosis as follows: OS (HR = 1.071, 95% CI: 0.526–2.186, P = 0.814), PFS (HR = 1.228, 95% CI: 0.668–2.255, P = 0.508), LRFS (HR = 1.284, 95%CI: 0.620–2.662, P = 0.500), and DMFS (HR = 1.772, 95% CI: 0.729–4.307, P = 0.202). Furthermore, Figure 3B shows a landmark analysis of OS at two time points within the first 5 years and beyond 5 years, based on whether chemotherapy was performed. The 5-, 10-, 15-year OS rate was 79.4%, 27.9% and 18.6% in the chemotherapy group versus 67.2%, 58.8% and 41.8% in the no chemotherapy group, respectively. During the initial 5-year follow-up period, OS did not differ significantly between the two groups; however, a significant difference emerged during in later follow-up periods, with poorer survival outcomes observed in the chemotherapy group.

### Acute toxicity

The chemotherapy group showed a higher incidence of hematologic-related events, both in grade 1-2 (P = 0.018) and grade 3-4 reactions (P = 0.036). Both arms had similar distributions of nausea/vomiting, mucositis, xerostomia, and kidney injury-related events (all P > 0.05). In both groups, the most common grade 1-2 adverse event was xerostomia, followed by mucositis and hematologic issues. There was no treatment-related fatalities (Table 3).

**TABLE 3:** Comparison of acute treatment toxicity in no Chemotherapy and Chemotherapy groups.

Variables	No chemotherapy (n = 37)		Chemocherapy (n = 50)		p1	p2
	Grade 1/2	Grade 3/4	Grade 1/2	Grade 3/4		
Nausea/vomiting	5 (13.5%)	0	10 (20.0%)	0	0.428	NA
Hematologic	15 (40.5%)	0	33 (66.0%)	6 (12.0%)	0.018	0.036
Mucositis	16 (43.2%)	3 (8.1%)	24 (48.0%)	5 (10.0%)	0.66	1.0
Xerostomia	30 (81.0%)	4 (10.8%)	42 (84.0%)	6 (12.0%)	0.722	1.0
Kidney injury	0	0	3 (6.0%)	0	0.258	NA

NA = not applicable.

## Discussion

Adjuvant chemotherapy in head and neck adenoid cystic carcinoma (ACC) is typically reserved for advanced-stage disease. However, there is a paucity of studies exclusively focusing on ACC originating from the paranasal sinuses. Due to the aggregation of data across various anatomical sites in most head and neck ACC studies, it is difficult to draw specific conclusions about SNACC. Although the chemotherapy might have a palliative benefit (Laurie et al., 2011), its long-term survival effects remain unclear, particularly in T4-stage SNACC. To address this critical clinical question, we conducted a retrospective study evaluating the role of platinum-based chemotherapy in T4-stage sinonasal adenoid cystic carcinoma.

Despite the frequent occurrence of late recurrence and distant metastasis, SNACC patients may still exhibit prolonged survival, with a 5-year OS rate of 55%–70% (details seen in Supplementary Table S2) (Lupinetti et al., 2007; Unsal et al., 2017; Akbaba et al., 2019; Trope et al., 2019; Jang et al., 2025; Hu et al., 2020). However, this rate drops significantly to 40% at 10 years and further decreases to 15% by 20 years (Husain et al., 2013; Mays et al., 2018). Previous research has identified the T4 stage as an independent prognostic factor associated with decreased local control in SNACC patients (Michel et al., 2013; Akbaba et al., 2019). T4 stage tumors frequently involve surrounding organs at risk, such as orbit, cranial nerves, dura, cavernous sinus, carotid arteries, and brain, limiting the possibility of radical resection (Unsal et al., 2017). The involvement of the organ at risk makes treatment challenging. Additionally, pathological subtypes are also closely associated with patients’ prognosis. Classic ACC often shows a combination of cribriform and tubular patterns. In most studies, a solid growth pattern is a predictive factor for poor prognosis, advanced stage and development of distant metastases (Ferrarotto et al., 2021). Clinically, managing T4 lesions represents a significant therapeutic dilemma. In our study, despite receiving multidisciplinary treatment, up to 25.3% of patients still experienced local recurrence, and 14.9% faced distant metastasis. Consistent with the literature (Husain et al., 2013; Mays et al., 2018), the 5-, 10-, and 15-year OS rates for T4 stage SNACC were 74.3%, 47.6%, and 33.0%, respectively. During the first 5 years of follow-up, the results suggested a high survival rate. However, the OS and PFS rates were shown to decrease drastically during the subsequent follow-up periods, which may be attributed to the high rate of later recurrence and distant metastasis.

Chemotherapy plays a limited role in ACC, primarily used for patients with unresectable, recurrent or metastatic disease (Dillon et al., 2016). Chemotherapeutic agents such as Cisplatin, Adriamycin, and Vinorelbine (CAP) can be used as monotherapy or in combination chemotherapy schedules (Rentschler et al., 1977; Dodd and Slevin, 2006; Vermorken et al., 1993; Ross et al., 2009). CAP is the most commonly used combination chemotherapy regimen; however, it is not regarded as a standard treatment due to the balance between its high toxicity profile and treatment efficacy (Papaspyrou et al., 2011; Ha et al., 2020). Hyerim et al. (Ha et al., 2021) observed that concurrent chemoradiation with cisplatin was effective for local control and had tolerable toxicity in ten cases of locally advanced unresectable ACC. In our study, patient with orbital/nasopharyngeal/cavernous sinus involvement were prone to receive platinum-based chemotherapy as a component of their treatment (Table 1). A combination of cisplatin, doxorubicin, and cyclophosphamide was the most common chemotherapy regimen (46%, 23/50). Unfortunately, logistic regression analysis indicated that chemotherapy could not reduce the risk of distant metastasis in both univariate (P = 0.383) and multivariate analyses (P = 0.213). Pooled data from studies on ACC indicate that chemotherapy demonstrates a low objective response rate (0%–30%), and stable disease (SD) and symptomatic relief are more commonly achieved outcomes (Papaspyrou et al., 2011; Dodd and Slevin, 2006). In the Surveillance, Epidemiology, and End Results (SEER) database, Micha et al. (Trope et al., 2019) found that surgery, but not radiation therapy or chemotherapy, predicted better OS for SNACC patients. In Figure 3, during the univariate Kaplan-Meier curve analysis, no statistical difference was observed between the chemotherapy group and the non-chemotherapy group, and the curves showed a crossing phenomenon. Thus, we adopted landmark analysis and found that there was no statistical difference in 5-year OS between the two groups (P = 0.080). However, as the follow-up time extended, patients in the chemotherapy group exhibited worse long-term survival. The lower 10-year OS (P = 0.034) in the chemotherapy group may be due to the toxic effects of chemotherapy and its tendency to favor patients with less favorable prognostic indicators (orbital/nasopharyngeal/cavernous sinus involvement) who were difficult to achieve negative surgical margins. Risk factors such as positive lymph nodes (N^+^), orbital involvement, and nasopharyngeal invasion often influence treatment decisions, including the use of chemotherapy (Megwalu and Sirjani, 2017; International and Neck Scientific, 2016; Qazi et al., 2016). Our subgroup analyses indicate that chemotherapy does not provide a survival benefit among specific populations, such as those with N^+^, orbital invasion or nasopharyngeal invasion.

This study has several limitations that require consideration. The primary weakness of our research was that it was a single-center retrospective study, which introduced the potential for selection and information bias. For instance, the choice of whether to administer chemotherapy may have been influenced by prognostic factors that were not included in the multivariate analysis, highlighting the need for prospective studies, that are currently lacking. Additionally, prognosis is associated with histologic subtypes (i.e., tubular subtype, cribriform or solid), but the absence of data regarding histological classification made it impossible to perform a subgroup analysis. Despite these limitations, to our knowledge, this is the only reported series of patients with T4 stage SNACC treated with chemotherapy.

## Conclusion

In this retrospective analysis, platinum-based chemotherapy did not seem to provide a long-term survival advantage for patients with T4 SNACC and might be associated with worse outcomes after 5 years, although these findings need confirmation through prospective studies.

## Data Availability

The original contributions presented in the study are included in the article/Supplementary Material, further inquiries can be directed to the corresponding authors.
