# Supplementary figures and images for: Long-term outcomes of platinum-based chemotherapy for T4 stage sinonasal adenoid cystic carcinoma
Source: Front Pharmacol. 2025 Sep 29;16:1623242. doi: 10.3389/fphar.2025.1623242 (PMC12515957; doi:10.3389/fphar.2025.1623242)

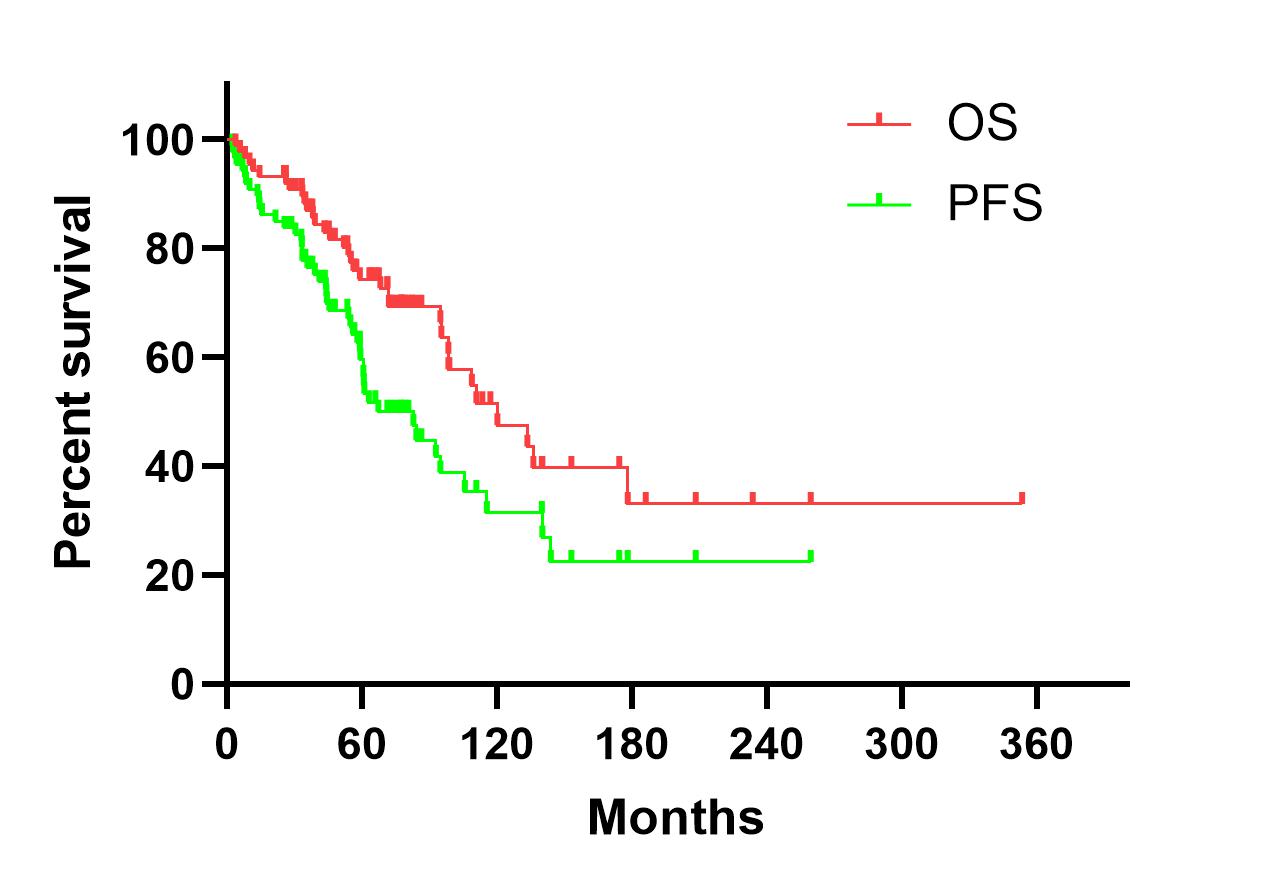

Supplement: Supplementary file 1 [file Image1.jpeg]
