# Supplementary material for: Long-term outcomes of platinum-based chemotherapy for T4 stage sinonasal adenoid cystic carcinoma
Source: Front Pharmacol. 2025 Sep 29;16:1623242. doi: 10.3389/fphar.2025.1623242 (PMC12515957; doi:10.3389/fphar.2025.1623242)
Supplement: Supplementary file 2 [file Table1.doc]

**Supplemental Table 1. Chemotherapy summary of patients**

| Chemotherapy Summary | No. of patients | Percent(%) |
| --- | --- | --- |
| Chemotherapy regimen |  |  |
| Platinum + Cyclophosphamide + Doxorubicin | 23 | 46.0 |
| Platinum + Docetaxel + 5-fluorouracil | 3 | 6.0 |
| Platinum + 5-fluorouracil | 11 | 22.0 |
| Platinum + Gemcitabine | 1 | 2.0 |
| Platinum | 12 | 24.0 |
| Chemotherapy cycles |  |  |
| 1-2 cycles | 40 | 80.0 |
| 3-5 cycles | 10 | 20.0 |

Platinum: cisplatin/ nedaplatin
