# Supplementary material for: Long-term outcomes of platinum-based chemotherapy for T4 stage sinonasal adenoid cystic carcinoma
Source: Front Pharmacol. 2025 Sep 29;16:1623242. doi: 10.3389/fphar.2025.1623242 (PMC12515957; doi:10.3389/fphar.2025.1623242)
Supplement: Supplementary file 3 [file Table2.docx]

| **Supplementary Table 2. Studies of sinonasal adenoid cystic carcinoma** | | | | | | | | | |
| --- | --- | --- | --- | --- | --- | --- | --- | --- | --- |
| Year | N | Follow-  up (m) | T3/T4 | N0 | M0 | Treatment | LCR | DM rate | OS |
| 2007 | 105 | 47.0 | 76.7% | 98.0% | 97.0% | 84.0% (S+R) | 30.0% | 38.0% | 62.9% (5-y) |
| 2017 | 694 | NS | 75.0% | 96.4% | 92.9% | NS | NS | NS | 64.5% (5-y) |
| 2019 | 227 | 50.0 | 95.2% | 87.7% | 98.7% | C-ion | 26.9% | 31.4% | 79.0% (3-y) |
| 2019 | 793 | NS | 48.4% | 96.4% | 96.3% | 77.4% (S) | NS | NS | 61.0% (5-y) |
|  |  |  |  |  |  | 68.2% (R) |  |  |  |
|  |  |  |  |  |  | 16.4% (C) |  |  |  |
| 2020 | 38 | 27.2 | 94.7% | 97.4% | NS | PBT/C-ion | NS | NS | 96.7% (3-y) |
| 2025 | 19 | 87.5 | 47.3% | NS | NS | 58.8% (S+R) | 35.3% | 36.4% | 63.0% (3-y) |
|  |  |  |  |  |  | 23.5% (S+CCRT) |  |  |  |
|  |  |  |  |  |  | 5.9% (S) |  |  |  |
|  |  |  |  |  |  | 5.9% (CCRT) |  |  |  |

S: surgery; R: radiation therapy; C: chemotherapy, CCRT: concurrence chemoradiotherapy; PBT: proton beam therapy; NS: not stated; LCR: local control rate; DM: distant metastasis; OS: overall survival
